# Teachable Moments: Development of an Environmental Health Behavior Change Tool for Pregnant Women and Parents

**DOI:** 10.3390/ijerph23050674

**Published:** 2026-05-20

**Authors:** Rebecca H. Ofrane, Stella Agolli

**Affiliations:** Department of Public Health, Montclair State University, Montclair, NJ 07043, USA; agollis1@montclair.edu

**Keywords:** environmental health, pregnancy, health promotion, digital health

## Abstract

**Highlights:**

**Public health relevance—How does this work relate to a public health issue?**

- The perinatal period represents a critical window of susceptibility where environmental exposures, such as endocrine-disrupting chemicals and air pollution, can significantly impact long-term maternal and child health outcomes.
- While pregnant individuals are highly motivated to reduce risks, they often face a “knowledge–action gap” due to the complexity and overwhelming nature of environmental health data.

**Public health significance—Why is this work of significance to public health?**

- This study introduces a novel digital health tool that translates high-level federal datasets and evidence-based screening into a simplified health index to improve environmental health literacy.
- By utilizing Motivational Interviewing principles, the tool shifts away from traditional fear-based risk communication toward an empowerment-based model that fosters self-efficacy.

**Public health implications—What are the key implications or messages for practitioners, policy makers and/or researchers in public health?**

- Digital screening tools that provide personalized, low-cost behavioral recommendations can effectively scale environmental health interventions beyond clinical settings.
- Leveraging objective, localized data can help address environmental justice concerns by identifying risks in historically underserved or red-lined communities.

**Abstract:**

The perinatal period is a critical window of susceptibility for fetal development and awareness for women’s health. Pregnant women are highly motivated to reduce environmental health risks, yet often lack personalized, actionable guidance on mitigating endocrine-disrupting chemicals and other household hazards. Grounded in Motivational Interviewing theory, a digital assessment was developed to empower parents to identify and reduce exposures. The tool screens for home-based and environmental risks across several domains: air quality, lead, tobacco, cleaning agents, pesticides, and plastics (BPA/phthalates). Based on user inputs, a defined algorithm generates a positive index score paired with prioritized, low-cost behavioral recommendations designed to shift users from risk awareness to active mitigation. Since its launch in Spring 2024, the tool has had over 1900 views. Preliminary analytics suggest promising engagement, and feedback more so suggests that the motivational-interview-based framing, which emphasizes empowerment over fear, facilitates immediate behavioral changes, such as switching to safer personal care products and improving indoor ventilation. Digital health interventions that translate complex environmental data into a single, manageable score can bridge the gap between clinical knowledge and household practice. This article details the score’s calculation methodology and underlying datasets, and reports usage analytics and user feedback, discussing how digital screening can scale environmental health literacy and improve maternal and child health outcomes.

## 1. Introduction

The perinatal period is a critical window of susceptibility for fetal development, during which environmental exposures can have lasting impacts on both maternal and child health outcomes [1]. During this time, pregnant individuals are often highly motivated to adopt healthier behaviors to protect their developing child, making it an important opportunity for intervention. However, despite this motivation, many expectant parents lack clear, personalized, and actionable guidance on how to reduce everyday environmental exposures within their homes [2].

Environmental risks such as endocrine-disrupting chemicals, including compounds like bisphenols and phthalates, are commonly encountered in daily life and have been linked to adverse reproductive and developmental outcomes [3]. While awareness of these risks has increased, translating complex environmental health information into practical, behavior-changing guidance remains a challenge [4]. Prior research has shown that prenatal environmental health education can improve awareness and promote protective behaviors, but many existing approaches are either too generalized or difficult for individuals to apply in their daily lives [4,5].

Technology-driven approaches have the potential to address these gaps. Digital health interventions, including mobile applications and structured online education modules, have demonstrated potential in helping pregnant women reduce environmental exposures by providing tailored, user-friendly information [6,7]. At the same time, tools that simplify complex health data into scoring systems have been shown to improve user engagement and understanding by translating information into a more accessible and actionable format [8,9]. These approaches highlight the importance of combining personalization and simplicity along with behavioral theory to effectively support behavior change. 

In addition to knowledge gaps, research suggests that how environmental health information is delivered plays a critical role in whether individuals take action. Many pregnant individuals report feeling overwhelmed by the volume and complexity of environmental risk information, which can lead to confusion or inaction rather than behavior change [4]. At the same time, studies show that women are indeed interested in receiving this information and prefer guidance that is practical, personalized, and easy to implement in their daily routines [2]. Interventions that incorporate behavioral frameworks, such as Protection Motivation Theory and Motivational Interviewing, have been shown to be more effective in supporting behavior change by emphasizing self-efficacy, autonomy, and achievable steps rather than fear-based messaging [5]. These findings highlight the importance of designing tools that not only inform, but also actively support individuals in making realistic and sustained changes. 

Building on this growing body of work, there remains a need for user-centered tools that not only increase awareness but also guide individuals toward meaningful action. The digital assessment described here was developed to address this gap by providing a personalized evaluation of environmental exposures within the home. The tool generates a simplified score along with tailored recommendations designed to support behavior change. By leveraging the heightened motivation that occurs during pregnancy and early parenthood, this approach aims to move individuals from awareness to actionable risk reduction, ultimately improving maternal and child health outcomes. This article details the score’s calculation methodology and underlying datasets, and reports the usage analytics and user feedback, discussing how digital screening can scale environmental health literacy and improve maternal and child health outcomes.

## 2. Materials and Methods

### 2.1. Assessment Development and Theoretical Framework

The assessment was developed as a digital environmental health behavior change tool specifically for pregnant individuals and parents. The assessment’s design is grounded in Self-Determination Theory (SDT) and Protection Motivation Theory, and operationalized through Motivational Interviewing (MI). SDT posits that motivation and behavior change depend on fulfillment of 3 innate psychosocial needs: autonomy, competence and relatedness [10]. MI developed as a clinical communication style designed to strengthen a patient’s intrinsic motivation toward health behavior change. It is related to SDT through 4 key elements: partnership (i.e., relatedness), acceptance (i.e., autonomy), compassion and evocation (competence and relatedness) [11]. While MI is traditionally a clinical technique used to foster medical action [10], it is increasingly applied as a standalone theory of action for behavioral health change [12].

The assessment domains (air quality, lead, tobacco, cleaning agents, pesticides, and plastics) were selected based on high-priority household toxicants identified in established maternal and child health environmental health literature. Questions and response options were designed to ensure they remained actionable for a non-technical audience. The MI framework is operationalized through a person-centered, empathic interface that avoids fear-based messaging [11]. Rather than prescribing behavior, the tool aims to resolve ambivalence by identifying the user’s existing protective behaviors and offering actions for further risk reduction, thereby supporting the user’s autonomy and self-efficacy.

The tool adopts an empathic, person-centered approach, avoiding the confrontational or fear-based messaging often found in environmental risk communications. To support the SDT constructs of autonomy and competence, the assessment is designed to provide actionable feedback that allows users to choose their own path toward risk reduction. Protection Motivation Theory supports this approach by emphasizing how individuals assess both perceived risk and their ability to respond effectively, which can strengthen motivation to engage in protective health behaviors [5,13]. The teachable moment of pregnancy and early parenthood is leveraged by focusing on the user’s heightened motivation to protect fetal and infant health, and to reduce toxic chemicals potentially transporting through the placenta to the developing fetus.

### 2.2. Survey Design and User Experience

The assessment consists of nine questions designed to maintain a low respondent burden, adhering to survey methodology best practices for digital engagement [14]. The flow of questions follows a macro-to-micro logic, starting with broad environmental settings such as air quality and county-level lead risk, moving to the home environment such as cleaning products and pesticides, and ending with personal behaviors including as personal care products and plastics to encourage intrapersonal reflection. The topics assessed include potential exposures to toxic chemicals via lead risk, air pollution, tobacco smoke, cleaning products, pesticides in the home or workplace, chemicals in plastics, pesticide residue on food, volume of personal care product use, and ingredient-checking behaviors.

### 2.3. Scoring Algorithm and Data Integration

The resulting “BetterNest Score” employs a standardized algorithm to convert complex environmental data into a 1–10 positive health index. In alignment with MI principles, higher scores represent lower environmental risk and higher protective behaviors. Objective risk for lead and air quality is calculated using the respondent’s zip code to identify county-level data from federal sources.

For lead exposure risk, data is retrieved from the CDC’s National Environmental Public Health Tracking Network [15]. A risk score is assigned based on the normal distribution quintiles of the percentage of housing built before 1980 in the user’s county. 2020 American Community Survey data was used to establish the following quintile thresholds: greater than 69.3% of housing built before 1980 (assigned a score of 1); 58.8–69.3% (score of 3); 50.3–58.8% (score of 5); 41.1–50.3% (score of 7); less than 41.1% (score of 9).

For air pollution (PM_2.5_), risk is calculated from the monitored and modeled particulate matter concentration of the user’s county, as determined by their zip code. Scoring tertiles are anchored to the EPA’s 2020 National Ambient Air Quality Standards (NAAQS) [16]. A score of 9 is assigned for annual average standard below 12 µg/m^3^; a score of 5 assigned to concentrations between 12 and 15 µg/m^3^, while levels exceeding 15 µg/m^3^ receive a score of 1. See Table 1.

All other exposures are scored based on risk-adjusted distributions of respondent answers. A specific logic is applied to personal care products: Question 8 (volume of product use) does not contribute to the final average. Instead, it serves as a modifier for Question 9 (ingredient screening), treating the volume of use as a modifying multiplier that contextualizes the impact of the user’s protective screening behaviors. The final score is calculated as an unweighted arithmetic mean of the scores from eight domains, with Question 8 excluded. As this is not meant as a formal chemical exposure risk assessment, but as an informational tool, an unweighted approach was chosen to ensure the tool remains interpretable for the target audience of new or expectant parents. The resulting mean is rounded to the tenths to provide a score from 1.0 to 10.0. Actual score calculations can range from 1.3 to 9.4, an intentionally limited range designed to prevent a perfect score of 10 (leaving room for improvement) or an exact score of 1 (so as not to demoralize or instill fear in users).

Upon completion, respondents receive their final score along with their top three actionable recommendations for exposure reduction. These recommendations are automated based on the user’s highest-risk responses: the three lowest-scoring questions (lower scores represent more risk) are used for personal recommendations. Examples of recommendations include:

- “There might be a risk of lead exposure in your home. Consider testing your tap water, and look into ways to reduce lead in the home. Get recommendations here (link to factsheets).”
- “Try to reduce ingestion of plastics. Avoid heating food or drinks in plastic, and switch to metal, glass or other materials for products where possible. Get more recommendations here (link to factsheets).”
- “Keep trying to reduce the number of personal care products you use daily, or check their ingredients and swap out for safer products based on your priorities. Get more recommendations here (link to factsheets).”

All feedback is framed within MI theory, utilizing non-judgmental language to encourage autonomy and self-efficacy.

## 3. Results

**Figure 1:**
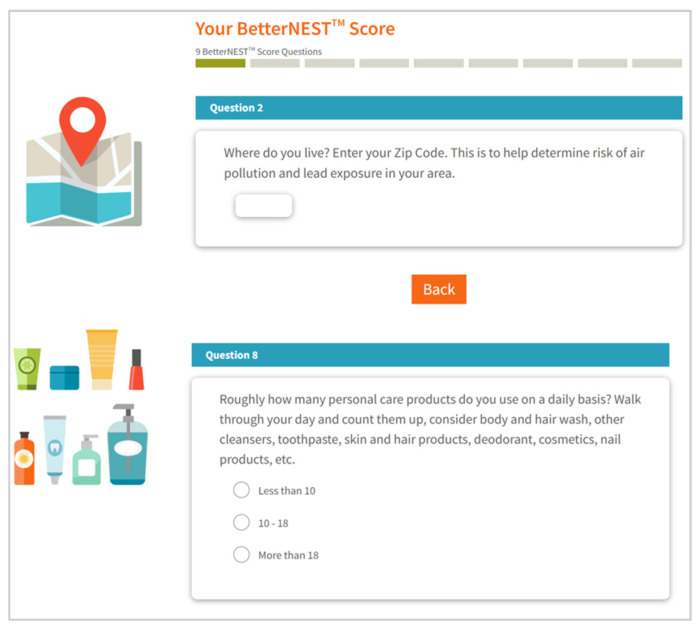
BetterNest Assessment Screenshots of Questions 2 and 8.

The assessment—known as the BetterNest Score—launched on the existing SafetyNest website in Spring 2024 [14] (mySafetyNest, LLC, El Cerrito, CA, USA). See Figure 1 for screenshots of selected assessment questions from the website, demonstrating the graphic visual language. The digital assessment takes less than 5 min to complete, and is itself designed as a learning experience, in addition to users receiving a final score and recommendations.

Site analytic data reveal that the BetterNest Score and assessment see approximately 85 views per month on average, for a total of almost 1900 views as of March 2026. A usability testing focus group of users and members of the target audience was held in Spring 2025, with nine participants. Participants were recruited through SafetyNest’s social media, and included a mix of birth professionals (doulas and childbirth educators) and current and expecting parents (some of the birth professionals were also parents themselves). The focus group included time for participants to explore the website and complete the assessment, and prompts for discussion and feedback (see Appendix A for prompts).

Feedback from the focus group indicated that the assessment was “easy”, “efficient” and “thought-provoking”, and was the appropriate length for a busy mom or pregnant woman who may have a low tolerance for friction. One user suggested the questions felt “obvious”, indicating that tool’s utility may vary depending on a user’s baseline environmental health literacy. Suggested improvements included adding (1) an explanation of why these questions and topical areas were selected for the assessment, and (2) a summary of which potential exposures contribute most to the respondents’ score, in addition to the top three standardized recommendations already provided.

## 4. Discussion

The development of the BetterNest Score represents a novel application of digital health technology to bridge the gap between complex environmental toxicology data and actionable maternal health behaviors. By leveraging the “teachable moment” of pregnancy, the tool translates high-level datasets—such as the EPA’s National Ambient Air Quality Standards and CDC housing metrics—into a personalized health literacy instrument. This approach aligns with the growing need for decentralized, accessible public health tools that address the social and environmental drivers of health at the household level.

While the BetterNest Score does not serve as a clinical diagnostic for chemical exposure, its content validity was established through preliminary review by birth professionals and environmental health researchers to ensure the domains aligned with current perinatal risk data. Concurrent validity (the degree to which this tool aligns with established assessments) was considered by anchoring objective scoring for lead and air quality to federal CDC and EPA datasets. Future validation efforts will focus on measuring behavior change and estimated exposure reductions based on scoring results.

While scoring and recommendations do not incorporate race, the tool is designed to address equity in a few ways. First, a user’s zip code identifies their county for associated air pollution and lead in housing exposures, both often linked to environmental justice concerns in communities with less resources for advocacy, and previously red-lined neighborhoods [17,18]. Second, higher daily volume of personal care products has been linked to higher rates of exposure in non-Hispanic Black women [19].

The integration of Motivational Interviewing (MI) into the scoring feedback is a critical departure from traditional risk communication, which often relies on fear appeals that can lead to fatalism or avoidance within pregnant populations. By framing the results as a positive health score and providing autonomy-supportive recommendations, the BetterNest Score fosters a sense of competence and empowerment. This is particularly vital in environmental health, where individuals often feel overwhelmed by the ubiquity of potential toxins like PM_2.5_ or microplastics.

### Strengths, Limitations and Future Directions

The focus group feedback suggests that the macro-to-micro question flow and the short assessment length are effective for the target demographic of busy parents. The demand for more detailed breakdowns of which exposures contribute most to the final score indicates user interest and a desire for deeper environmental health literacy.

A strength of the BetterNest methodology is its use of localized, objective data via zip code identification. This simplifies the cognitive demand from the user, who may not know the age of their local housing stock or the specific particulate matter concentrations in their county. Another strength is the applicability of the quiz to a diverse audience, focused on the target audience of non-technical parents, but those who are engaged in the concern of environmental health exposures.

There are also limitations to both the tool and this pilot evaluation. First, relying on county-level data specifically for air quality and lead exposure estimation may mask hyper-local environmental injustices or exposures within a single zip code. Future iterations of the tool could benefit from more granular, census-tract level data to improve the precision of the lead and air quality risk assessments, or applicability to global audiences.

Another limitation of this study is the small size of the focus group (*n* = 9), which may not fully represent the diversity of the target population. Future research should involve a more rigorous, longitudinal evaluation with a larger, diverse cohort to assess the tool’s impact on objective health behaviors and to potentially validate the score against environmental biomarkers. This would allow for a more definitive assessment of the tool’s ability to shift lifestyle behaviors and resulting exposure reduction across different socioeconomic contexts.

As the tool continues to scale, we also aim to collect more detailed user analytics data for understanding which environmental concerns are most prevalent among modern parents, potentially informing future state and local public health policy and training.

## 5. Conclusions

The “BetterNest Score” serves as a scalable model for how environmental health behavior change can be incentivized through user-centered design and digital health tools. Preliminary analytics suggest promising engagement with the personalized recommendations. While initial user feedback indicates that the motivational-interview-based framing may facilitate self-reported behavioral changes, these findings are descriptive and based on a limited pilot sample. Further research is required to determine the long-term sustainability of these behaviors. By centering the user’s autonomy and providing a clear, quantified pathway to exposure reduction, the tool moves beyond mere awareness of personal environmental exposures toward the actualization of protective health behaviors during a critical window of human development and behavior change.

## Figures and Tables

**Table 1:** Questions, Possible Responses and Scoring Criteria.

Question	Responses	Associated Scores
Q1. Are you considering pregnancy, currently pregnant, or have a baby or young children at home?	I’m considering pregnancy	N/A
	I’m currently pregnant	
	I have a baby or young children at home	
Q2. Where do you live? Enter your zipcode. This is to help determine risk of air pollution and lead exposure in your area.	No response. Estimates lead risk, or percentage of housing built before 1980, by county	<41.05 = 9
		41.05–<50.3 = 7
		50.3–<58.8 = 5
		58.8–<69.3 = 3
		>69.3 = 1
	No response. Estimates air quality, historical PM_2.5_ monitored and modeled, by county	<12 = 9
		12–15 = 5
		>15 = 1
Q3. How often are you exposed to tobacco or other smoke?	Never, or only by passing smokers outdoors in public places	10
	Sometimes, I’m around smokers a few times a week, mostly outdoors	6
	Frequently, someone in my home smokes	3
	Daily, I smoke	1
Q4. How often are you exposed to conventional cleaning products (consider home and at work)? [For those considering or currently pregnant]. How often are conventional cleaning products used in your home? [For those who have a baby/child at home].	Almost never, I only use “green” products with minimal chemical ingredients	9
	Weekly, or a few times a month	6
	Daily, or a few times a week	2
Q5. How often are pesticides sprayed in or around your home or building?	Never or rarely	9
	Sometimes, or I’m not sure	5
	Regularly, monthly or more frequently	1
Q6. How often do you drink from, eat from, or heat food or drink in plastic containers? [For those considering or currently pregnant]. How often does your child drink or eat from plastic containers, drink or eat items heated in plastic, or chew on plastic toys? [For those who have a baby/child at home].	Almost never	10
	A few times a month	7
	A few times a week	3
	Daily	1
Q7. How often do you buy organic produce for you or your family, or check the pesticide level on the DirtyDozen list?	Always, or at least the ones recommended by DirtyDozen	9
	Sometimes	5
	Never, I buy what’s available or cheapest	2
Q8. Roughly how many personal care products do you use on a daily basis? Walk through your day and count them up, consider body and hair wash, other cleansers, toothpaste, skin and hair products, deodorant, cosmetics, nail products, etc.	Less than 10	9
	10–18	5
	>18	1
Q9. How often do you check the ingredients of your or your child’s personal care products on a tool like SkinDeep, Think Dirty, or Clearya?	All the time, for all the products I can	If Q8 = 9, then 10; If 5, then 7; If 1, then 5
	Sometimes	If Q8 = 9, then 8; If 5, then 6; If 1, then 3
	Never	If Q8 = 9, then 7, if 5, then 3, if 1, then 1

## Data Availability

The original contributions presented in this study are included in the article. Further inquiries can be directed to the corresponding author.

## Abbreviations

The following abbreviations are used in this manuscript:

CDC	U.S. Centers for Disease Control and Prevention
EPA	U.S. Environmental Protection Agency
PM_2.5_	Particulate Matter, 2.5 microns
SDT	Self Determination Theory
MI	Motivational Interviewing
NAAQS	National Ambient Air Quality Standards

## Appendix A. Usability Testing Focus Group Discussion Prompts

SafetyNest—Usability Focus Group

June 2025

1. To start, could you introduce yourselves? We have a mix of doulas and parents, so please share your first name, whether you’re a doula and/or parent, and why you’re interested in this topic.
2. Please take a few minutes to explore the Safetynest website on your own, and take the BetterNest quiz. Feel free to click around, explore different sections, and take the quiz either as yourself, or if you’ve already taken it, try entering different answers this time.
3. As you explore, please try to think about: What stands out? What’s easy to use? What’s confusing? What questions come to mind?
4. When you first landed on the website, what were your immediate thoughts or feelings? What was the first thing that caught your eye?
5. On a scale of 1 to 10 (1 being very easy, 10 being very difficult), how easy or difficult was it to navigate and find the information that was helpful?
6. What did you find most challenging or frustrating?
7. What words come to mind about your interaction with the BetterNest quiz?
8. Were the questions clear and straightforward?
9. Did the quiz’s results or recommendations make sense to you? Was the output easy to understand?
10. What, if anything, would make the quiz more effective or user-friendly?
11. What next steps or additional information would you want after taking the quiz, or what kind of follow-up?
